# Transarterial embolization in the management of chronic shoulder pain: clinical evidence and emerging therapeutic role

**DOI:** 10.3389/fradi.2026.1753020

**Published:** 2026-06-10

**Authors:** Nicolò Ubaldi, Federico Virgili, Federico Perconti, Aleksejs Zolovkins, Gianluigi Orgera, Edoardo Ronconi, Miltiadis Krokidis, Michele Rossi, Marcello Andrea Tipaldi

**Affiliations:** 1 Department of Surgical and Medical Sciences and Translational Medicine, Sapienza-University of Rome, Rome, Italy; 2 Department of Interventional Radiology, Sant’Andrea University of Hospital La Sapienza, Rome, Italy; 3 National and Kapodistrian University of Athens—Aretaiio Hospital, Aretaieion Panepistemiako Nosokomeio, Athens, Greece

**Keywords:** adhesive capsulitis, chronic shoulder pain, interventional radiology, rotator cuff tendinopathy, transarterial embolization, trapezius myalgia

## Abstract

Chronic shoulder pain is a prevalent and disabling condition encompassing diverse pathologies such as adhesive capsulitis, tendinopathy, enthesopathy, and trapezius myalgia. These disorders share multifactorial mechanisms involving chronic inflammation, angiogenesis, and neurogenic sensitization, leading to persistent pain and functional limitation. Recent insights into the vascular basis of shoulder pain have highlighted pathological neovascularization as a potential therapeutic target. This narrative review explores the role of transarterial embolization (TAE) as a minimally invasive treatment option for chronic shoulder pain refractory to conservative management. Adhesive capsulitis, characterized by capsular fibrosis and restricted motion, demonstrates significant pain reduction and improved range of motion following TAE, with technical success rates approaching 100% and minimal adverse events. Similarly, rotator cuff tendinopathy and trapezius myalgia have shown marked improvement in pain and function, confirming the role of TAE in disrupting pathological neovascular and neurogenic pathways. Various embolic agents, including imipenem/cilastatin sodium, polyethylene glycol, and calibrated microspheres, have been used with favorable safety profiles, though concerns regarding antibiotic resistance warrant further evaluation. Overall, current evidence supports TAE as a safe, effective, and joint-preserving alternative to surgery for patients with chronic shoulder pain of vascular-inflammatory origin. While the literature demonstrates consistent improvements in pain scores and functional outcomes, large-scale randomized controlled trials remain necessary to validate these findings and establish standardized treatment protocols.

## Introduction

Chronic shoulder pain encompasses various pathological entities, among which are rotator cuff injuries, adhesive capsulitis (AC), glenohumeral osteoarthritis, tendinopathy, and enthesopathy. These syndromes have a multifactorial etiology that integrates systemic and biomechanical factors (1). The pathogenic mechanisms of these diseases involve chronic inflammation, proliferative fibrosis, angiogenesis, and neurogenic pain. Recent evidence suggests that hypervascularity in the rotator interval and overexpression of vascular endothelial growth factor promote neovascularization and substance P-positive nerve proliferation, sustaining chronic pain. This condition arises from a self-perpetuating cycle of inflammation, angiogenesis, and fibroblastic proliferation (2, 3).

Chronic shoulder pain is a global burden, affecting between 10.8% and 55.2% of the population. Women report shoulder pain more frequently than men (4). The prevalence of shoulder pathology increases after the age of 50 years among employed individuals, with a significant economic impact on both healthcare systems and society (5). In 2000, the direct cost burden for the treatment of shoulder dysfunction in the United States was $7 billion (6).

This pain syndrome is frequently sustained by a pathological hypervascularization of the soft tissues involved, characterized by the proliferation of abnormal microvessels and accompanying nociceptive nerve fibers, which contribute to persistent pain and restricted motion. Unlike the knee or hip joint, the shoulder is not a weight-bearing joint, and, therefore, mechanical deterioration, including cartilage loss and axial degeneration, is less common; as a result, vascular-inflammatory modulation through embolization becomes a more rational therapeutic target. The vascular anatomy of the shoulder—dominated by well-defined branches of the thoracoacromial, circumflex humoral, and subscapular arteries—allows for a precise embolization, minimizing the risk of non-target ischemia.

Interventional radiology (IR) has recently offered alternatives to treat different musculoskeletal disorders of the shoulder joint (7). In the last few years, there has been an initial interest in IR in the management of patients presenting with musculoskeletal pain in general, including the shoulder joint; this is perhaps explained by the fact that interventional radiologists can provide minimally invasive treatments directly to the target area, with rapid, safe, and effective clinical results. Transarterial embolization (TAE) has demonstrated the ability to disrupt pathological inflammatory pathways, thereby representing a promising minimally invasive therapeutic option for these conditions. Its application may also contribute to reducing the excessive use of analgesics and intracapsular injections of steroidal drugs commonly required in patients with chronic pain, with a consequent reduction in all the important associated side effects.

This narrative review aims to offer a systematic approach to the conditions that most commonly underlie chronic shoulder pain that is refractory to standard conservative treatment and which may benefit from IR TAE procedures. Through this prism, the pathophysiology, indications, and techniques are summarized in conjunction with the expected results and procedure safety.

## Adhesive capsulitis

### Background

AC, a condition also known as frozen shoulder, is characterized by progressive pain and restriction of both active and passive glenohumeral motions. The underlying pathology involves synovial inflammation, capsular thickening, and fibrosis, resulting in pain and mechanical limitations of movement. The prevalence of AC is 2%–5%, predominantly affecting middle-aged individuals over 40 years of age, with female predominance (8). Secondary adhesive capsulitis may also develop following shoulder trauma, fractures, rotator cuff injuries, or surgical interventions (9).

AC progresses through four stages (10). The initial phase presents with gradual shoulder pain, often nocturnal, and mild glenohumeral motion limitation. The freezing stage combines persistent pain with increasing capsular contracture and restricted movements. In the third, or frozen stage, stiffness becomes the predominant symptom. The final thawing stage is marked by minimal pain and progressive recovery of motion.

The clinical diagnosis of adhesive capsulitis is based on persistent shoulder pain and limitation of both active and passive motion for >3 months, in the absence of alternative causes (11). However, because of the overlap of symptoms with other shoulder disorders, clinical differentiation may be difficult, highlighting the pivotal role of imaging in precise evaluation and treatment planning.

Magnetic resonance imaging is a valuable modality for assessing soft tissue inflammatory changes and confirming the diagnosis of adhesive capsulitis (12). More specifically, rotator interval and axillary capsule enhancement demonstrate the highest sensitivity, and coracohumeral ligament thickening provides the greatest specificity for diagnosis (13). Recent advances in imaging have proposed the incorporation of the superb microvascular imaging ultrasound technique into diagnostic protocols, potentially enabling earlier detection of frozen shoulder and improving patient prognosis (14).

### Clinical management

An evidence-based systemic model for the medical management of AC has not yet been established. AC is generally managed through a stepwise approach, beginning with conservative therapy and progressing to minimally invasive or surgical options in refractory cases (15). Physiotherapy remains the first line of treatment, often combined with non-steroidal anti-inflammatory drugs (NSAIDs) or short courses of corticosteroids for pain control. Intra-articular corticosteroid injections and hydrodilatation provide short-term symptomatic relief by reducing inflammation and stretching the contracted capsule. The majority of patients have their symptoms resolved spontaneously after 1–3 years of conservative treatment (16). Suprascapular nerve block may further enhance pain control (17). Finally, surgical approaches are reserved for persistent cases refractory to conservative treatment, including manipulation under anesthesia (MUA) and arthroscopic capsular release (ACR) (18). Both ACR and MUA have demonstrated significant improvements in shoulder pain and functional outcomes (19).

Despite these therapeutic options, approximately 35% of patients with AC continue to experience mild-to-moderate symptoms, and 6% report severe symptoms after 4 years (20). For patients unresponsive to these measures, TAE has recently emerged as a novel minimally invasive potential option targeting abnormal pericapsular neovascularization. The primary indication for TAE is shoulder pain for at least ≥3 months that is refractory to conservative management, although studies have reported results involving patients with shoulder pain unresponsive to a minimum of 30 days of conservative therapy (21).

### Treatment technique

**Figure 1:**
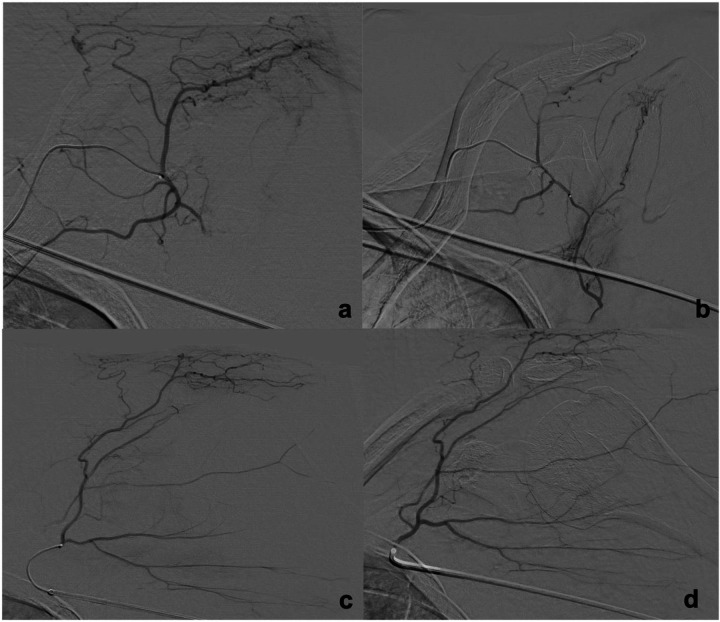
Angiographic series of shoulder images before and after TAE in a 62-year-old man with frozen shoulder. **(a,c)** Selective angiography of the humeral circumflex and thoracoacromial artery before embolization shows abnormal distal vessels with contrast medium blush of the soft tissue. After embolization, the distal blush is no longer visible **(b,d)**.

**Figure 2:**
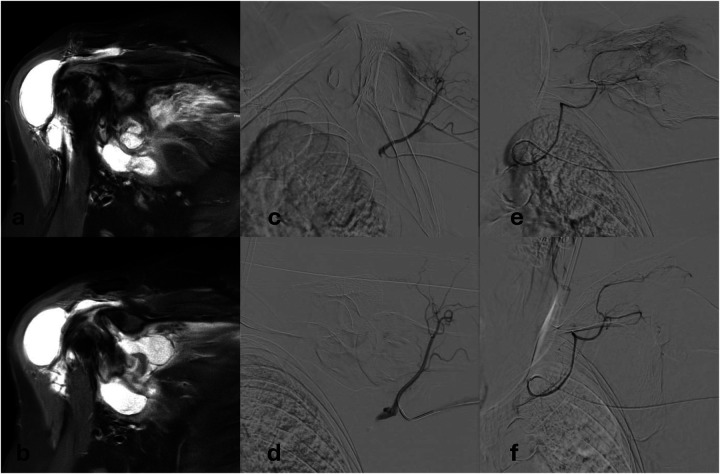
MRI before the procedure shows a 54-year-old man affected by a condition of distension and inflammation of the capsular-ligamentous complex of the shoulder, extending to the superior, anterior, and inferior recesses, in addition to bursitis of the sub-acromial deltoid, sub-coracoid, and subscapular bursae **(a,b)**. Angiographic series of shoulder images before and after TAE **(c–f)**. Selective angiography of pathologically inflamed arteries before embolization, characterized by contrast medium blush of the soft tissue **(c,e)**. After embolization, the distal blush is no longer visible **(d,f)**.

TAE is usually performed under local anesthesia, with additional reports using topical ice packs to minimize undesired migration of the embolic agent (22, 23). Vascular access can be achieved via the radial or femoral artery under ultrasound guidance, using a 4/5 French (F) sheath. With a coaxial technique, using a diagnostic 4/5F catheter and a microcatheter 1.7–2.7F in size, with a compatible microwire 0.012–0.018 inches in size, depending on the operator's preference and expertise, the humeral circumflex, subscapular, suprascapular, and thoracoacromial arteries are super-selectively catheterized. Iodine contrast is systematically injected under fluoroscopy guidance from the microcatheter to identify abnormal neovascularization, typically described as hyperemic regions exhibiting a “blush” enhancement pattern with early venous drainage (24), as shown in Figures 1, 2. The procedural goal is generally a selective and conservative reduction of pathological neovascularity rather than complete arterial stasis or aggressive devascularization. In the majority of reported series, operators aimed for the reduction or disappearance of the abnormal hypervascular blush while preserving flow within the parent artery and adjacent normal vascular territories. Cone beam CT (CBCT) can be used during shoulder embolization, although most published series on embolization for shoulder pain syndromes rely primarily on digital subtraction angiography (DSA) for target vessel identification, without routine use of CBCT. DSA findings include abnormal periarticular hypervascularity, pathological neovessels, vascular blush, and/or early venous return. When CBCT is available and technically feasible, it can be used as an adjunct for three-dimensional assessment of arterial anatomy and for excluding any non-target vessels. Moreover, CBCT is helpful at the end of the procedure to document the anatomical localization of the injected particles and exclude any off-target deposition. The majority of studies used resorbable embolic particles, specifically imipenem/cilastatin sodium (IMP/CS) (22, 24–31). Alternatively, polyethylene glycol microspheres (HydroPearl™, Terumo, Tokyo, Japan) have been employed (21, 32); however, to date, only Gremen et al. have published results with calibrated non-resorbable microspheres measuring 100–250 µm in size (Embozene®, Boston Scientific Corporation, Marlborough, MA, USA) (23).

All patients were discharged within 2–8 h following TAE, except in the study by Ciampi-Dopazo et al. (29), where discharge occurred the following day. Post-procedure, the majority of authors recommend relative rest for the day, with gradual resumption of shoulder movement beginning from the next day. Patients were advised to continue their pre-existing conservative therapy but were restricted from initiating any new treatment modalities for 6 months after the intervention (33).

### Results

Following TAE for AC, all the studies reported a rapid reduction in pain intensity, as measured by the visual analog scale (VAS) or numeric rating scale (NRS), along with progressive improvement in shoulder range of motion (ROM). Fernández et al. reported that 70% of patients achieved a VAS score ≤3 at 1-month follow-up, while at baseline, 70% of patients reported a pain score of 8 and 28.6% reported a score of 10. A significant improvement in ROM was also documented, with shoulder flexion increasing from 79.5° to 133° at 6 months post-procedure (22). Okuno et al. (24) found that 88% of patients were satisfied with the procedure at 6 months. Non-resorbable microspheres were associated with moderate efficacy, with 20% of patients reporting ≥50% pain reduction at the 3-month follow-up (23).

Supportive results have also been reported in the management of secondary adhesive capsulitis. TAE was performed in 25 patients with postoperative or post-traumatic shoulder stiffness unresponsive to at least 3 months of conservative therapy (28). A significant pain reduction (8 vs. 2 VAS score, *P* < .001) and shoulder mobility improvement (70° vs. 150° ROM; *P* < .001) were observed at 6-month follow-up. The results are summarized in Table 1.

**Table 1:** Summary of TAE outcomes according to pain scores (VAS or NRS).

Study	Disease	Study design	Patients (n)	Follow-up time	VAS-NRS pre-TAE	VAS-NRS post-TAE
Okuno et al. (25)	AC	Prospective, non-randomized	7	6–16	67 ± 14	2 ± 5
Okuno et al. 2016	AC	Prospective, non-randomized	25	30–44	68 ± 14	1 ± 4
Fernandez-Martinez et al. 2020	AC	Prospective, non-randomized	40	12–48	8	2
Sajan et al. (32)	AC	Case report	1	6	83	13
Fernandez-Martinez et al. 2022	AC	Retrospective	25	6–41	8	1
Bagla et al. (21)	AC	Prospective, non-randomized	20	6	89	62
Gremen et al. (23)	AC	Retrospective	15	6	7 (7–8)	5 (3–7)
Okuno et al. (24)	AC	Prospective, non-randomized	76	6	6 ± 2	2 ± 2
Liang et al. (30)	AC	Retrospective	25	6	7 ± 2	1 ± 1
Fernandez-Martinez et al. (27)	AC	Retrospective	118	6	8	2
Okuno et al. (45)	RTC	Retrospective	7	4	73 ± 10	10 ± 7
Hwang et al. (46)	RTC	Retrospective	13	4	6 ± 1	3 ± 2
Kim et al. (47)	RTC	Prospective, non-randomized	10	1	4 ± 1	2 ± 2
Shibuya et al. (49)	TM	Retrospective	42	6	9 ± 1	4 ± 3

Data are presented as mean ± standard deviation.

VAS, visual analog scale; NRS, numerical rating scale; FU time, follow-up in months; AC, adhesive capsulitis; RTC, rotator cuff tendinopathy; TM, trapezius myalgia.

A 100% overall technical success rate was achieved, defined as selective catheterization and embolization from at least one artery feeding the shoulder joint.

### Safety

Across all studies, only minor adverse events were observed, including groin discomfort related to arterial puncture-site hematoma, transient skin discoloration or erythema, self-limiting radial artery spasm, and short-term fever. No major complications were observed. The incidence of ischemic events was lower with IMP/CS than with non-resorbable embolic materials. This reduction can be attributed to the formation of micro-sized, temporary embolic particles by the IMP/CS-iodinated contrast mixture, which are reabsorbed within 1–48 h (25, 34). However, the widespread use of IMP/CS could raise concerns regarding the emergence of antibiotic-resistant microorganisms, which could limit its suitability for daily clinical practice (35). Moderate adverse events for non-resorbable microspheres were related to off-target embolization, including post-embolization syndrome, transient paresthesia, temporary humeral bone marrow oedema, and transient skin necrosis (23).

## Enthesopathy and tendinopathy

### Background

Tendinopathy involves a spectrum of tendon disorders, primarily classified as tendinitis and tendinosis. Tendinitis represents an acute inflammatory response, typically resulting from microtears within the tendon structure, whereas tendinosis reflects a chronic degenerative process of collagen fibers associated with repetitive mechanical stress and overuse (36).

The underlying mechanisms of tendinopathy and enthesopathy are not clearly defined, and they are often considered self-limiting conditions. Various treatment approaches have been proposed to manage these conditions, including eccentric exercise programs, anti-inflammatory medications, extracorporeal shockwave therapy, and injections such as corticosteroids or platelet-rich plasma, sclerotherapy, growth factor treatment, and surgery (37, 38). Studies have shown that neovascularization and associated nerve growth are potential contributors to pain in these conditions (39, 40). Rotator cuff tendinopathy is frequently associated with functional impairment of the upper limb, restricting daily activities and significantly diminishing quality of life (41).

### Clinical management

An evidence-based standardized management of rotator cuff tendinopathy has not yet been established. Exercise is widely recommended as a first-line therapeutic approach. Extracorporeal shockwave therapy and non-steroidal anti-inflammatory drugs have demonstrated short-term pain relief but not significant improvement in shoulder function scores (42). Laser therapy has shown limited efficacy in improving pain in the short term (42). Platelet-rich plasma injections have been associated with significant reductions in pain at medium- (6 months) and long-term (12 months) follow-up, though they offer no short-term (3 weeks) benefit and do not significantly enhance shoulder function (43). Despite conservative management, nearly 30% of patients with tendinitis and bursitis remain symptomatic after 6 months (44).

The primary indication for TAE is persistent shoulder pain for at least ≥3 months despite conservative management (45), in some cases reaching >6 months (46), with or without intratendinous calcific deposits (47).

### Treatment technique

The treatment technique is based on the same foundational approach as that described previously for AC.

### Results

Okuno et al. (45) were the first to introduce TAE for the treatment of musculoskeletal tendinopathy. A significant reduction in pain scores was observed at the 4-month follow-up (72.7 mm ± 9.9 mm vs. 9.7 mm ± 6.8 mm). Except for a single case of self-limiting subcutaneous hemorrhage, no procedure-related complications were observed.

Building on these findings, Hwang et al. treated 15 cases of shoulder or elbow tendinopathy in 13 patients. Additional studies using Embospheres or IPM/CS have demonstrated significant improvement of VAS scores at 4 months post-embolization (6.1 ± 1.3 vs. 2.5 ± 2.0) (46). However, the study's conclusions regarding the efficacy of TAE for shoulder tendinopathy remain limited, as the outcomes were analyzed across the entire cohort without stratification by etiology.

To address this limitation, a prospective study enrolled 10 patients with rotator cuff tendinopathy who had persistent pain unresponsive to conventional therapies and were treated with TAE using IPM/CS (47). Significant improvements in VAS and Shoulder Pain and Disability Index (SPADI) scores were observed from baseline to 1 day and 1 month after treatment (VAS score 3.5 ± 0.9, 2.00 ± 1.33, and 1.70 ± 1.25, respectively; SPADI 38.46 ± 14.15, 20.23 ± 14.98, and 18.69 ± 12.32, respectively). The results are summarized in Table 1.

### Safety

No major complications occurred, further supporting the safety and effectiveness of TAE demonstrated in previous studies (46, 47).

## Trapezius myalgia

### Background

Trapezius myalgia is a condition characterized by acute or chronic neck and shoulder pain associated with stiffness and tightness of the upper trapezius muscle and is most frequently observed in individuals who perform repetitive manual tasks or maintain prolonged, strained postures (48).

### Clinical indication

Cases of trapezius myalgia for more than 6 months and refractory to conservative management have been treated with TAE using IPM/CS, targeting the transverse cervical, suprascapular, and circumflex scapular arteries (49).

### Treatment technique

The treatment technique is based on the same foundational approach as that described previously for AC and tendinopathies.

### Results

During the 6-month follow-up, patients demonstrated a significant reduction in pain intensity and improvement in physical function, achieving a clinical success rate of 71.4%. The remaining patients experienced either partial symptom relief or no improvement. A progressive reduction in the need for conservative therapies was observed over the follow-up period (49). The results are summarized in Table 1.

### Safety

Only one minor adverse event was observed, namely, mild subcutaneous hemorrhage at the puncture site, which resolved within 1 week. No major complications were reported (49), although the literature is limited.

## Therapeutic positioning of TAE and proposed clinical algorithm

**Figure 3:**
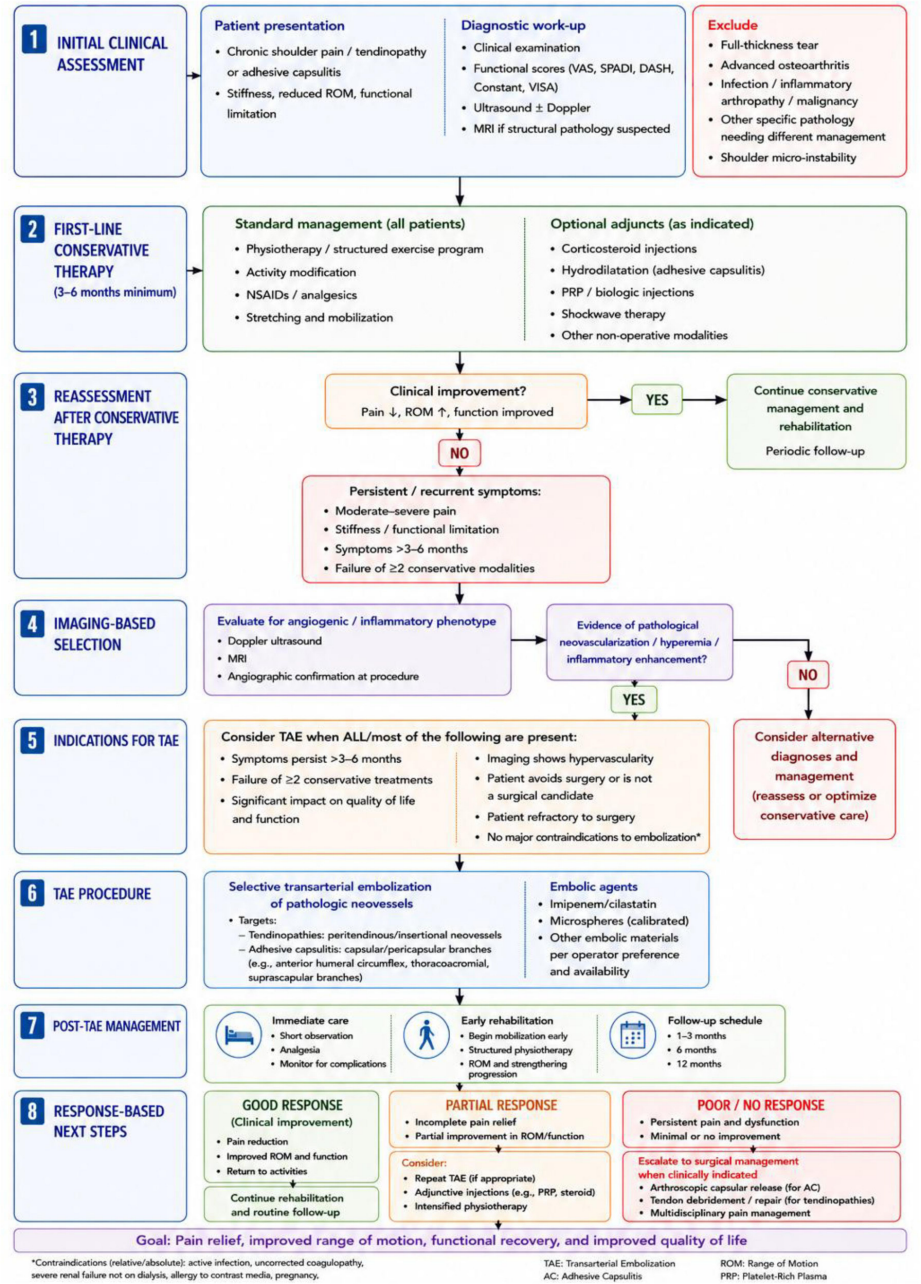
Proposed treatment algorithm for TAE in tendinopathies and adhesive capsulitis.

TAE should currently be considered an intermediate-line, minimally invasive treatment for refractory tendinopathies and adhesive capsulitis, positioned after failure of conservative therapies but before surgical intervention in selected patients, within a stepwise multidisciplinary care pathway. Contemporary evidence particularly supports its use in patients with persistent pain, functional limitation, and imaging evidence of pathological hypervascularity, while surgery remains reserved for cases unresponsive to both conservative management and embolization. TAE is increasingly emerging as a targeted intervention and may represent an earlier minimally invasive alternative to surgery in selected refractory patients, minimizing the risk of over-injection of corticosteroids and joint capsule/tendon destruction. Cases also refractory to surgery could be treated with TAE (Figure 3).

Given the lack of comparative trials, no embolic agent can currently be considered definitively superior; agent choice should be individualized according to target vascular anatomy, degree of hypervascularity, operator experience, and safety considerations. Temporary resorbable agents—most commonly imipenem/cilastatin—represent the predominant embolic strategy for adhesive capsulitis and related tendinopathies, consistent with the goal of transiently reducing pathological neovascularity while minimizing the risk of durable ischemic injury in periarticular soft tissues.

## Conclusions

Given the absence of a formal consensus on the optimal therapeutic management of AC, tendinopathies, and other musculoskeletal diseases of the shoulder joint, TAE has emerged as a safe and effective minimally invasive option for pain relief and restoration of shoulder mobility. There is an emerging necessity to promote and introduce minimally invasive IR musculoskeletal embolization procedures for shoulder pain management. While the current evidence supports its clinical potential, further rigorous, large-scale randomized trials with control interventions are necessary to support its application in clinical practice.
